# A General Odorant Background Affects the Coding of Pheromone Stimulus Intermittency in Specialist Olfactory Receptor Neurones

**DOI:** 10.1371/journal.pone.0026443

**Published:** 2011-10-18

**Authors:** Angela Rouyar, Virginie Party, Janez Prešern, Andrej Blejec, Michel Renou

**Affiliations:** 1 UMR1272, PISC, Institut National de la Recherche Agronomique - Université Pierre et Marie Curie, Versailles, France; 2 Department of Entomology, National Institute of Biology, Ljubljana, Slovenia; Freie Universitaet Berlin, Germany

## Abstract

In nature the aerial trace of pheromone used by male moths to find a female appears as a train of discontinuous pulses separated by gaps among a complex odorant background constituted of plant volatiles. We investigated the effect of such background odor on behavior and coding of temporal parameters of pheromone pulse trains in the pheromone olfactory receptor neurons of *Spodoptera littoralis*. Effects of linalool background were tested by measuring walking behavior towards a source of pheromone. While velocity and orientation index did drop when linalool was turned on, both parameters recovered back to pre-background values after 40 s with linalool still present. Photo-ionization detector was used to characterize pulse delivery by our stimulator. The photo-ionization detector signal reached 71% of maximum amplitude at 50 ms pulses and followed the stimulus period at repetition rates up to 10 pulses/s. However, at high pulse rates the concentration of the odorant did not return to base level during inter-pulse intervals. Linalool decreased the intensity and shortened the response of receptor neurons to pulses. High contrast (>10 dB) in firing rate between pulses and inter-pulse intervals was observed for 1 and 4 pulses/s, both with and without background. Significantly more neurons followed the 4 pulses/s pattern when delivered over linalool; at the same time the information content was preserved almost to the control values. Rapid recovery of behavior shows that change of perceived intensity is more important than absolute stimulus intensity. While decreasing the response intensity, background odor preserved the temporal parameters of the specific signal.

## Introduction

The localization of a sexual partner is a critical step in an insect's life and it often involves chemical signals [1]. In moths, males respond by an upwind flight to the specific pheromone blend emitted by the females [2]. The male moth must not only discriminate the pheromone in a complex odorant environment, but it must also be able to follow the intensity changes of the pheromone signal. Therefore detection of quality and temporal parameters of the pheromone signal and their transformation into a neuronal code by the olfactory system is important for controlling oriented flight behaviour [3]. Due to their chemical specificity, the pheromone receptors expressed in certain olfactory receptor neurones (ORNs) of the male antennae constitute a first-order filter for the pheromone signal, distinguishing the sex pheromone from other odours [4]. Pheromone-sensitive receptor neurones (Ph-ORNs) are specialized and narrowly tuned to one of the components of the pheromone blend [5]. They show remarkable discrimination capacities of their key compounds among isomers or other analogues and do not respond to general odorants. However, it is known for a long time that some volatile organic compounds interfere with pheromone detection in Ph-ORNs. Schneider et al. [6] showed that presentation of geraniol suppressed the response of Ph-ORNs to the natural pheromone in the moth *Antherea pernyi*. In turn, Ochieng et al [7] found that Ph-ORNs responses to blends of (*Z*)-11-hexadecenal, a primary pheromone component, and linalool or (*Z*)-3-hexenol, two common plant volatiles, were increased compared to the responses to the pheromone component alone. However, in other moth species, linalool and other terpenoids antagonize the pheromone when added to the pheromone source [8], [9], [10]. Similar effects on responses to pheromone pulses were observed in *Spodoptera littoralis* when linalool or geraniol were presented as an odorant background [11]. In *Heliothis virescens*, addition of a plant compound, which itself did not elicit a response in Ph-ORNs, typically suppressed the Ph-ORN responses to the pheromone, with a single exception for β-caryophyllene [9]. All together, these results indicate that pheromone detection by moth ORNs is influenced by other volatile compounds from the environment, and that the most frequent type of interaction between general odorants and pheromone is mixture suppression.

Under natural conditions, beside to the complexity created by the presence of numerous other odorants, the air turbulences distribute the pheromone molecules in time and space (reviewed in [3], [12]). Such conditions create a stream of pheromone pulses separated by gaps of clean air that meander downstream [13]. The temporal distribution of the pheromone pulses varies considerably in space. As distance from the source increases, odour plumes tend to spread, larger fluctuations are detected far downstream, and moths might use intermittency or cues derived from the shape of odour pulses to steer in direction of the odour source [12]. Male moths must therefore react quickly to successive encounters of the pheromone plume and prolonged disappearances by changing the direction of their flight. Their Ph-ORNs must encode a highly intermittent signal which changes in amplitude (low-high concentration) and rate (scarce-frequent) to provide reliable information to the male. In turn, the projection neurones in the antennal lobe generate discrete bursts of firing and there is a direct link between the bursting firing pattern of the PNs and the odour-tracking behaviour of the male moth [14]. The ability of Ph-ORNs to code for stimulus intermittency has been acknowledged as a critical point for olfactory orientation and was extensively studied. Single sensillum recordings showed that the Ph-ORNs of the moth *Antheraea polyphemus* were able to resolve 20 ms pheromone pulses up to 5 pulses/s (pps) [15]. The temporal resolution of Ph-ORNs depended upon the temperature, being only 2.5 pps at 18°C [16]. Likewise, in the sphingid moth *Manduca sexta* ORNs were able to follow 20 ms pulses at a rate of 3 pps [17]. Bau et al. [18], [19] showed by using Fourier transformation that in case of fused peaks, the firing activity was still modulated according to the stimulus intermittency. In experiments with moths belonging to four taxonomic families, periodic fluctuations in electroantennogram (EAG) recordings reproduced stimulus cadences as high as 25 pps. Even higher temporal resolution (up to 40 pps) was observed in cockroach for 1-hexanol [20], a plant compound with small molecular weight, which is more volatile than the components of moth pheromones. Barrozo and Kaissling [21] studied temporal resolution in various moth species and reported a strong decrease in ORN responses to the second and following odorant pulses (compared to the first pulse) when presenting pulse trains. This decreased response intensity across successive pulses may result either from sensory adaptation, which involves the alteration of the transduction pathway, or most simply from the intrinsic dynamics of the cell [16]. The responses of Ph-ORNs are generally considered as phasic-tonic [22], [23], with a progressive decline of the firing activity after the end of the stimulation. After the stimulus offset, it takes several hundreds of seconds before the activity of the ORN comes back to the pre-stimulus level depending on the cell type and the active compound [16], [21] resulting in a partial fusion of the firing responses to successive pulses. Thus, neuron dynamics might be the most important limiting factor at high stimulation rates. The dynamics of the odour stimulator devices used to deliver pulses is not always precisely described and it can also produce partially fused pulses contributing to reduce the intermittency of the stimulus. Finally, a decrease of firing response to successive pulses is still observed with inter-pulse intervals large enough to enable a return to the base level activity [21], [24] indicating that short term adaptation [25] may also indirectly limits the temporal resolution by reducing the sensitivity.

These studies of capacity of insect ORNs to follow stimulus periodicity have been carried out using clean air as a carrier for the odorant stimulus. However, in natural conditions such intermittent stimuli are detected in a complex odorant environment. The presence of a background odour has significant effect on the olfactory sensitivity in *Musca domestica* [26] leading to cross adaptation and cross sensitization. The responses of the Ph-ORNs of *S. littoralis* to 100 ms pulses of pheromone were shorter in a linalool background, compared to clean air [11] making the individual responses to pulses more salient. Both contributions suggest that general odours commonly present in a natural environment significantly alter the dynamics of the responses to a pulsed specific signal.

In this paper we test the hypothesis that a non-specific odorant (termed background odour) could also affect the ability of Ph-ORNs to code temporal parameters of the pheromone signal. To determine to which extent a background odour can affect temporal coding in Ph-ORNs we compared the responses to pulse trains of pheromone in clean air and in a linalool background. Linalool was chosen among other terpenes as the most effective compound in reducing response to pheromone [11]. Interestingly, isoprene, a dominant component of the volatile emissions of deciduous trees, non host-plants for *S. littoralis*, did not change responses to pheromone [27]. First, we report on the effects of a linalool background on male orientation to a pheromone source. Second, we describe the temporal properties of pulses delivered by our stimulation device with a photo ionization detector (PID). Then, we analyse the firing responses of Ph-ORNs to 50 ms pulses of pheromone in a neutral versus linalool background. We studied the responses of ORNs specifically tuned to the main component of the pheromone blend (*Z*,*E*)-9,11-tetradecadienyl acetate (Z9,E11-14:Ac) housed in the long trichoid sensilla on the antennae of male *S. littoralis* [28], [29]. Using methods specific for measuring the periodic characteristics of the firing and the information content of the activity of a neurone population we describe the effects of background on the capacity of Ph-ORNs to follow temporal fluctuations of a specific signal.

## Materials and Methods

### Insects

*S. littoralis* were reared in the laboratory on an artificial diet at 23°C, 60 to 70% relative humidity and under a L16:D8 photoperiod until emergence. Sexes were separated at the pupal stage and maintained in different climatic chambers under an inverted LD regime. Newly emerged adults were collected every morning. Male moths were provided with a 10% sucrose solution.

### Chemicals

The main component of the female pheromone of *S. littoralis*, (*Z*,*E*)-9,11-tetradecadienyl acetate (Z9,E11-14:Ac, CAS 50767-79-8) was synthesized in the laboratory (courtesy of Martine Letteré). Its purity, checked by gas chromatography, was >97% purity. Dilutions were prepared in hexane (>98% purity, CAS 110-54-3) from Carlo-Erba, (Val-de-Reuil, France).

Linalool (racemic, 97% purity, CAS 78-70-6), was purchased from Fluka Analytical (Sigma-Aldrich, L’Isle-d’Abeau, France). White mineral oil from Sigma (CAS 8042-47-5) was used to prepare volume to volume dilutions at 0.1, 1, and 10%.

### Photo ionization detector

To trace the olfactory stimulus at the output of the stimulus delivery systems we used a fast response miniature photo ionization detector (PID, Aurora Scientific Inc, Aurora, Canada). Z9,E11-14:Ac could not be detected due to its high ionization potential, which is above the energy of the PID lamp (10.6 eV). In turn, linalool was reliably detected so that we used it instead of pheromone. We followed the variations of concentration of linalool in air as a continuous stimulus or within trains of 10 or 50 ms pulses at 1 to 10 pps, using a 10% solution of linalool in mineral oil as source. The signal from the PID was digitized and stored in a computer using a DT9816 AD board (Data Translation, Marlboro, USA) controlled by custom routines written with Measure Foundry (Data translation). The amplitudes and decays of the PID signals were then measured and analysed with applications specifically developed in R (http://www.r-project.org/).

### Behaviour

A locomotion compensator (LC-300, SYNTECH, Hilversum, Netherlands) was used to record the movements of male *S. littoralis* in response to stimulation. The locomotion compensator is made of a 30 cm diameter sphere on top of which the male is placed. Insect movements at the top of the sphere are recorded by an infrared light-sensitive camera positioned overhead. The digitized images provide coordinates of the centre of gravity of the animal. Using this information the insect displacement is real-time compensated by rotating the sphere using two electrical motors placed orthogonally so as to keep the centre of gravity of the insect on top of the sphere. A virtual insect path is therefore obtained from the sphere rotation and stored as incremental X and Y coordinates. Experiments were done during the activity period of *S. littoralis*, 2 to 4 h into the scotophase with red light, at 22–24°C.

To prevent flying, moths were anesthetized with CO_2_ soon after their emergence and their wings were removed before the onset of the scotophase 24 hours before the test. Wingless males responded reliably to the pheromone by walking (Minoli et al, in preparation). Olfactory stimuli were delivered with a programmable olfactometer adapted from Party et al. [11]. Air coming from the building supply was charcoal-filtered and divided in 2 flows in a Y-connector (model P514, Upchurch Scientific, Oak Harbor, USA). One flow was humidified and connected to the main branch of the “stimulation tube” to serve as permanent flow (9.6 L/min). The second flow was divided in 4 flows of 700 ml/min using a 5-port manifold used as stimulus carriers (model P-115, Upchurch Scientific). Each of the 4 flows was connected to a miniature electro-valve (model LHDA1233115H, The Lee Company, Westbrook, USA) piloted with a multi channel Valve Bank programmer (AutoMate Scientific, Berkeley, USA). The output of each valve was connected to 4-ml glass vials closed by septum corks with polytetrafluorethylene (PTFE) tubing (1.32 mm ID, 20 cm L). Hypodermic needles (18G size) were inserted through septum to connect the PTFE tubing. The linalool vial contained 1 ml of solution in mineral oil at 0.1%. As for the pheromone source, 1 µL of the main pheromone component in hexane (1 µg/µL) was deposited in a piece of PTFE tube (1.32 mm ID, 15 mm L) to ensure a final deposit of 1 µg. After hexane evaporated, the tube was inserted on the input needle of the vial. The male was constantly bathed by the permanent air flow plus a neutral flow and at stimulus presentation the odorized air replaced the neutral flow. A TTL signal was used to synchronize the acquisition of the walking path with the stimulation program. The sources of stimulus were renewed daily. Contaminated air was removed from the set-up by an exhaust fan.

#### Stimulus sequences

Experiments started about 20 s after placing the insect on the locomotion compensator, which was exposed to carrier air. At time = 0 the pheromone flow and a neutral air flow were introduced in the carrier air and we started to record the walking track of the insect. After 1 minute, the neutral air flow was replaced by a linalool or pure mineral oil (neutral background) was turned on and the walking track was recorded for 1 more minute. As control for the effects of background alone, the walking activity was also recorded in presence of linalool delivered during 2 minutes.

#### Analyses of walking track

Walking paths were sampled in TrackSphere software (Syntech, Hilversum, Netherlands) at 10 Hz and saved as csv files. The raw data were imported into TRACKS, a custom-made application in R, down-sampled to 5 samples/s, filtered by application of a local polynomial regression fitting (Loess) [30] and analysed. We calculated the following variables:

activation  =  number of insects showing active locomotion. A male was scored as active when it walked continuously on at least 75 mm. This distance was calculated by adding up individual steps of more than 0.5 mm to eliminate non locomotory movements.
orientation  =  number of insects showing locomotion and whose mean angle of track path was comprised within 0.52 radians on both sides of the source direction (0±30 degrees).
mean upwind speed at t_i_: the mean over a series of several tracks of the net displacement per second along the source axis for all sampled time t_i_.
orientation index at t_i_ *:* oi = cos (θ) * ρ, where θ is the mean angle of a series of tracks and ρ the length of the mean vector at t_i._

To analyse the effects of linalool on pheromone-induced locomotion we statistically compared the values of these variables measured during three 20 s periods corresponding to the activity before the change of background (ie from t = 40 to t = 60 s), the activity immediately following the change of background (from t = 60 to t = 80 s), and the activity after change of background (from 80 to 100 s), using a two sided, paired, Wilcoxon test.

### Electrophysiology

One-day-old virgin males were used for electrophysiological studies. Males were briefly anesthetized with CO_2_ and restrained in a Styrofoam holder. One antenna was fixed with small strips of adhesive tape. Single sensillum recordings were obtained from trichoid hairs using tip recording [5]. These sensilla were sampled among the long trichoid hairs that have been shown to house one Ph-ORN tuned to Z9,E11–14:Ac. The sensilla tip was cut off using sharpened forceps. A glass microelectrode containing a saline was placed over the sensilla tip [31]. The reference electrode was made of a chlorinated silver wire inserted into the abdomen. The recording and reference electrodes were connected to a Neurolog preamplifier (Digitimer, Hertfordshire, UK). The signal was filtered (0.2 to 10 kHz) and amplified 1000 times. The electrophysiological activity was sampled at 10 kHz and 12 bit resolution with a Data Translation DT3001 analog to digital card.

#### Olfactory stimulation

The odorants were delivered with a programmable stimulator as described in Party et al [11]. Charcoal-filtered air was re-humidified and divided in 8 equal flows (220±10 ml/min) directed each to a 3-way miniature valve. Thus the flow could be directed to the glass vial containing the stimulus source by activating the appropriate valve. The connections were made using PTFE tubing (1.32 mm ID). For linalool the vial contained 1 ml of mineral oil solution at 1% of linalool. The pheromone (1 µl of hexanic solution at 10 µg/µl) was deposited into a section of PFTE tubing (1.6 mm ID; L = 20 mm) directly connected to the vial inlet. One ml of pure mineral oil was used as neutral background. Stimulus and clean air carrying tubes were assembled together in a metal tubing of 10 cm long to constitute the stimulation pencil. A plastic cone of P1000 pipette was placed at the output of the pencil to constitute a mixing chamber. The tip of the cone was focused on the antenna with a micromanipulator. As for behavior, programming of the electro-valves was performed using a multi channel Valve Bank synchronized with signal acquisition. The Ph-ORNs were stimulated by the main pheromone component, delivered as a train of 10 pulses of 50 ms duration either in neutral background (pure mineral oil) or with linalool background. We tested three stimulation rates: 1, 4 and 10 pps. The ORN activity was recorded for 45 s. The background (either linalool or pure mineral oil) started at 14.5 s and the first pulse of pheromone was presented at 15 s. Background presentation was stopped 1 s after the end of the last pheromone pulse. Control stimulations were made using ten 50-ms pulses of pure mineral oil in air at a repetition rate of 4 pps.

#### Calculation of the firing rate

Spike sorting and extraction of spike occurrence times from the recordings were performed using Awave [32]. Some recordings showed spiking of two cells, which differed by spike amplitude. Only spikes with large amplitude were modulated by Z9,E11-14:Ac. The spike occurrence times were analysed using custom-written R scripts (http://www.r-project.org/). Firing rate was calculated using the local slope of the cumulative function of spike times [33]. Calculation of the slope was done over a moving spike window of n-2, n+2 spikes (5 spikes). Thus, each spike was attributed a firing rate and its occurrence time. This enabled a convenient graphical display of the variation of the firing rate over time for each individual recordings and calculation of the maximum and minimum frequencies of firing in response to each pheromone pulse.

#### Measure of contrast

The resolution of individual pulses within trains by the Ph-ORNs depends on the relative difference of firing at the local peaks during the pulses and the level of nervous activity during the inter-pulses. Due to the recovery time of the ORNs, their firing activity during the interval periods may be high enough to partially fuse their responses to individual pulses. To appreciate how the resolution was altered by stimulus pattern and odorant background, we calculated the local contrast for each pulse as C in dB: 
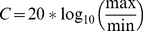
(1)


where max and min are respectively the maximum and minimum firing rates reached during each period of activity corresponding to a pulse and the following pulse interval.

#### Measure of response decay

The decay of response was fitted over an exponential asymptotic function, using the nls function of R. The firing rate during the response to the first pulse was measured in 54 and 45 recordings for the neutral and linalool backgrounds and averaged over time bin per time bin (bin size = 20 ms). The curves of the mean firing rates for the two backgrounds were standardized relatively to their respective maximum firing rate. The asymptotic decay functions were estimated from the time of the maximum firing rate to the end of the inter-pulse period: 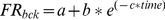
(2)


where FR_bck_ is the mean firing rate in neutral or linalool background, a is the offset, b the initial firing rate, and c the rate coefficient of the curve. The time values for a 90% decay (td_90_) were calculated from equation 2.

#### Temporal analysis

Following Barrozo and Kaissling [21], we used the Lomb-Scargle periodogram (LSP) to detect periodic pattern in the firing response of Ph-ORNs. The LSP method looks for significant periodic patterns in data time series. Compared to the methods commonly used to analyse periodic data, as the Fast Fourier Transform algorithm, the LSP can be used with data sampled on unevenly spaced time points and it is not affected by missing values. It calculates least-square fits to sinusoidal curves and derives the null distribution of the periodogram at a given frequency. A p-value of the periodogram is obtained using these results and the null hypothesis that data are not periodic versus the alternative that it is periodic can be tested. The LSP algorithm has been used to analyse various biological data and a software developed in R by Glyn et al for finding periodic gene expression profiles in microarray data [34]. We adapted the R code available at http://research.stowers-institute.org/efg/2005/LombScargle/R/index.htm to make it compatible with the format of our spike data. Searching for periodicity in firing data consisted of the following steps: (1) The firing rate for each recording and the mean firing rate over a series of recordings were calculated as described above. Because the stronger firing response to the first pulse could alter the calculation of the periodicity, only the activity from the second to the last pulse was considered. (2) The normalized Lomb-Scargle periodogram was calculated, and its peak determined. To avoid problems near the Nyquist limit, the frequency range for periodograms was restricted to a 0.5 to 20 Hz window. The value (Hz) of the dominant frequency peak and the probability (*p*-value) associated to that peak that it had occurred by chance (non-periodicity) was calculated. Neurons whose firing activity presented a dominant frequency equal to the period of the stimulus pulse rate±10% and a *p*-value≤0.05 were considered as following the stimulus periodicity. The number of follower neurons was calculated for each stimulation rate (1,4 and 10 pps) and each background (neutral, linalool).

#### Computation of Jensen-Shannon divergence

The global firing activity over a population of Ph-ORNs carries the information on the presence of pheromone. An increase in firing activity over base level correlated to pheromone pulse represents a gain in information and to optimally follow pulse trains the difference of firing between pheromone stimulated and unstimulated states should be maximum during, but minimum between pulses. To evidence the effects on the information contents of the firing activity due to the background odour we calculated the Jensen-Shannon divergence (D_JS_), a metric widely used in information theory (see [35] for an application to neurophysiology). The D_JS_ was used to measure the degree of overlapping in the distribution of spikes per bin between control and pheromone pulse trains. The divergence is maximum (D_JS_ = 1) when their distributions do not overlap, and the information content of the global activity is maximum. The value of D_JS_ decreases as the overlap between distributions increases. If the distributions overlap totally, the divergence is null, there is no information about the presence of an active odorant. Calculated bin by bin, the D_JS_ provides a measure of the information flux during pulses and in the intervals between pulses. For an optimal representation of the stimulus train, the D_JS_ should be large during pulses but small during inter-pulse intervals. We calculated the D_JS_ of firing responses to pulsed pheromone and the corresponding spontaneous firing recorded from the same neurones using custom-written scripts in R 2.10. From individual spike time series, the spike count within bins of 20 ms was recorded. Bin by bin response ensembles were constructed based on the distribution of spike counts for each bin. Between 12 and 20 responses were considered. Then, the program calculated the probability distribution of the spike counts (*i* = 0, 1, 2, 3,…, spikes) within the t^th^ 20 ms-bin. The probability of observing *i* spikes in the response ensemble p is p_t_(*i)*. The D_JS_ between the t^th^ bin of two spike trains, each with a spike probability distribution of pt and qt, respectively is:
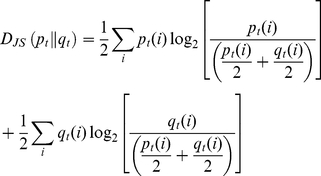
(3)


We then, compared the D_JS_ curves obtained from 57 recordings of responses to pheromone pulse at 4 pps in a neutral background and 46 in a linalool background.

## Results

### 1- Effect of linalool on walking behaviour

**Figure 1:**
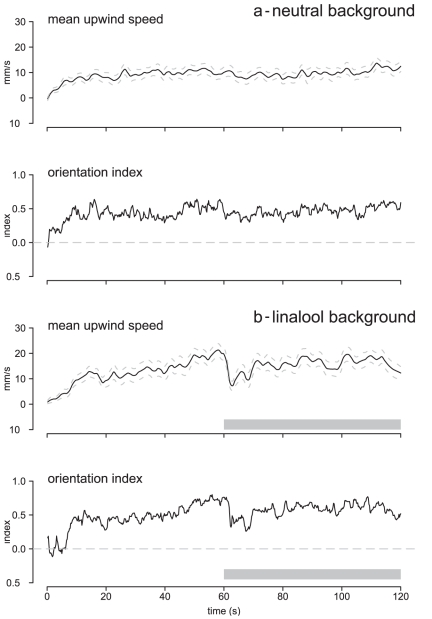
A linalool background alters orientation of male *Spodoptera littoralis* to pheromone source. Walking activity and orientation behaviour of male *Spodoptera littoralis* were measured on a locomotion compensator. Males were submitted to the main pheromone component, Z9E11–14:Ac during 2 min, ***1a:*** a neutral background was maintained for the duration of the test (n = 30). ***1b***: in addition to pheromone, a linalool background was applied after 1 min (n = 42). Mean upwind speed: average of the distances per second walked by males in direction of the source (solid line) and mean ± SEM (dashed lines). Orientation index: oi = cos (θ) * ρ, where θ is the mean angle of track sample and ρ the length of the mean vector at t_i._ Horizontal grey bar = linalool background.

The walking behaviour of male *S. littoralis* was recorded while they were stimulated with pheromone during two minutes, and the background was kept neutral, or was changed for linalool during the second minute. In a neutral background during the 2 minutes, 62% of the males were active and 48% of them oriented in direction of the stimulus source. The males active during the 1^st^ minute maintained their locomotion during the 2^nd^ minute of the test (Figure 1a).

During the tests with pheromone in neutral background followed by a linalool background, upwind speed and orientation index decreased immediately at onset of linalool presentation (Figure 1b). The upwind speed, averaged over 20 s periods, significantly decreased from 17.4 mm/sec before the change of background to 13.5 mm/sec immediately after change of background (*P* = 0.0089). Similarly, the orientation index decreased from 0.64 to 0.50 (*P* = 0.010). This decrease was temporary, as upwind speed and orientation index were not significantly different during the 20–40 s period after change of background compared to before the change (*P* = 0.179 and 0.142, respectively).

As control, we tested stimulation with linalool alone during 2 minutes. Only 12% of the males were active and no male oriented in direction of the source.

### 2- Characterization of the stimulus

#### Stimulus shape over the sphere in the locomotion compensator

**Figure 2:**
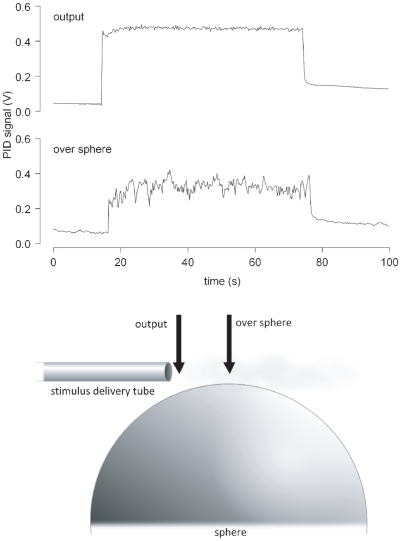
Monitoring of the odorant in the walking track recording device with a miniature Photo Ionisation Detector. To check the shape of the odour panache in the locomotion compensator, the probe of the PID was positioned either at the output of the stimulus delivering tube or over the sphere while a flow of air odorized with linalool (10% in mineral oil) was delivered.

We first measured the temporal pattern of odorant concentration in air as it arrives over a walking male. We positioned the PID either at the output of the stimulus delivering tube, or at 100 mm of the output and 5 mm over the sphere. Although being stable when measured directly at tube output, the PID response to a continuous 1 min pulse of linalool was jagged and non uniform over the sphere (Figure 2). The coefficient of variation of the amplitude of PID signal reached 88%. This confirmed that stimulus integrity quickly deteriorates within a few centimetres from the exit of our system as it happens with other stimulus delivery systems [36]. Thus, walking males were submitted to large variations of odorant concentration, even with a sustained stimulation.

#### Duration of pulses in the electrophysiological set-up

We then measured the PID responses to single pulses of linalool with different duration (10, 30, 50, 70, and 100 ms) to test the dynamics of the stimulus delivery system and select optimal pulse duration. The amplitudes of the PID signals clearly depended on the stimulus duration for pulses shorter than 100 ms. For 10 ms pulses, the amplitude of the PID signal was only of 15% of its value in response to 100 ms pulses, but it reached 71% at 50 ms pulse duration. At short stimulus times, only a fraction of the stimulus was delivered because of the time necessary for the air flow to carry the odorant from the vial to the output of the stimulator. To estimate this travel time of odorized air we measured the latency of the PID signal. The PID signal started to increase 44±8 ms after the TTL signal indicating the opening of the valve in the stimulation channel, and it reached its maximum amplitude at 82 ms±13 (n = 34). This result confirmed that the length of the tubing reduces the dynamics of the stimulator. However, such a length was imposed because it was necessary to use vials of odorant of a sufficient volume to produce constant stimulation level without exhausting the source, which could not been brought closer to the antenna due to space limitation in the electrophysiological set-up. In addition, separate tubing for each compound was preferred in order to avoid contamination and assure fast change in background. Thus, we chose 50 ms pulse duration as a compromise between duration short enough to achieve high repetition rates, but long enough to deliver enough odorant to get clear responses from the olfactory organs.

#### Reproducibility of pulses in pulse trains

**Figure 3:**
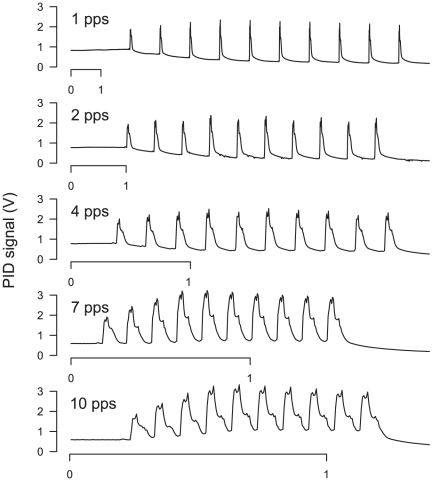
Responses of the PID to pulse trains of linalool. To trace the dynamics of the odorant concentration in air from the stimulation device in the electrophysiological set-up, the PID signal was recorded while linalool was delivered as ten 50 ms pulses at 1, 2, 4, 7 and 10 pps.

We then evaluated whether our olfactory stimulator was able to deliver constant odour pulses at different pulse rates (1, 2, 4, 7 and 10 pps) using trains of ten 50 ms-pulses. At all repetition rates, the PID signal showed individual peaks for each odour pulse (Figure 3). Then we measured the amplitude of individual peaks at every repetition rate. The amplitude of the PID signal significantly increased from the first to fourth pulses whatever the rate of the pulses. At 1, 2, and 4 pps the amplitude of the PID signal was constant from the fourth to the last pulse (ANOVA, *P*>0.09). At 7 and 10 pps, a 15% to 21% decrease was observed between the 4^th^ and the 10^th^ pulse (*P*<0.04). We attribute the lower intensity of the first pulses to the time necessary to progressively fill up the tubing between source and stimulator output. In turn, there was no “fatigue” of the source, the stimulus delivery system being able to reproducibly deliver pulses of the same intensity at repetition rates up to 4 pps.

With 7 and 10 pps the PID signal did not return to its base level during the inter-pulse interval. To estimate the speed of decrease of the odorant concentration after a pulse we measured the fall time of the PID signal within the pulse train at 1 pps (see material and methods for method of estimation). The 90% fall time for each pulse within pulse train at 1 pps was estimated to 150±0.03 ms (means and SD of n = 30 pulses). Thus, above a repetition rate of 4 pps, the inter-pulse interval was too short to allow the odorant concentration to come back to its base level, so the pulse train was composed of peaks of odorant concentration above a plateau.

### 3- Responses of Ph-ORNs to pulse trains

**Figure 4:**
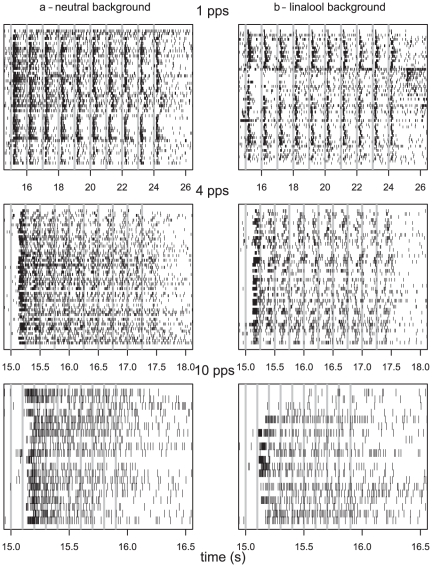
Ph-ORNs fire in synchrony with pheromone pulse trains in a neutral or linalool background. Raster plots of the firing activity of Ph-ORNs in response to pheromone stimulation by 10 pulses of 50 ms duration at 1, 4, and 10 pps (grey vertical bars mark stimulus onset) in neutral background (3a, n = 54, 57 and 20, respectively) and in linalool background (3b, n = 63, 45 and 20 respectively). Each line presents individual recording from a Ph-ORN localized in a long trichoid hair.

**Figure 5:**
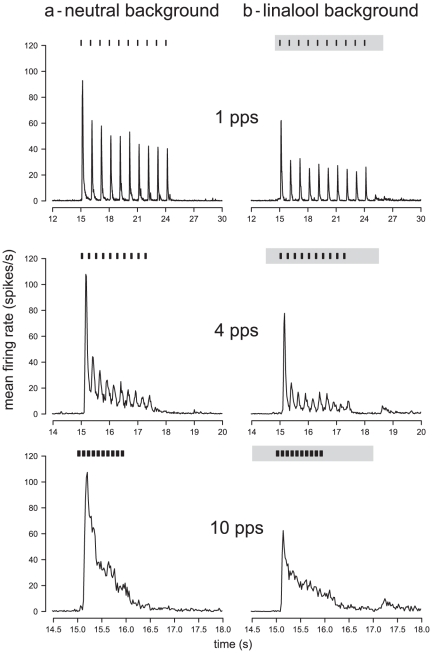
Background and pulse rate affect the firing response of Ph-ORNs to pulses. The curves presents the mean firing rates of the Ph-ORNs whose individual responses appear in figure 3 responding to pulse trains at 1, 4 and 10 pps in neutral (4a) and in linalool backgrounds (4b).

**Figure 6:**
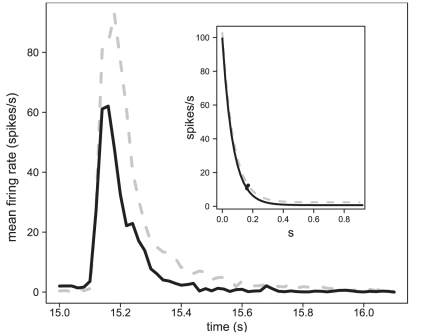
Effects of a linalool background on the decay of the firing response to a single pheromone pulse. The mean firing rate of Ph-ORNs in response to the 1st pulse of a pheromone pulse train at 1 pps in neutral background (dashed line, mean of n = 54 recordings) or linalool background (solid line, n = 45) was calculated. Then, we estimated the exponential decay function in neutral (inset: dashed line) or linalool background (solid line) in normalized scale; black points in the inset: 90% fall decay.

When presented with a train of ten 50 ms-pulses of pheromone at 1 pps in a neutral background, Ph-ORNs responded to the individual pulses by increasing the firing rate at each pulse (Figures 4a and 5a). Individual ORNs showed differences in their ability to follow stimulus pulses. The response to individual pulses was phasic-tonic (Figure 5a). The decay of the response to the 1^st^ pulse was fitted to an asymptotic decay function [eq 2] (Figure 6, dashed line). From this function we calculated a value of 170 ms for the 90% fall time. Then, we calculated the mean firing rate at inter-pulses by measuring the minimum firing rate in the 10 pulses of 11 experiments. The mean minimum firing rate, 3.4±0.7 spikes/s (mean±SEM), was significantly larger than it was before stimulus trains (1.2±0.2 spikes/s; Student's t test, *P* = 0.04). Thus, at 1 pps the firing activity of the Ph-ORNs did not come back to its resting level between pulses, but it did not impede the resolution of individual pulses by Ph-ORNs. The response to the first pulse was stronger than to subsequent pulses (Figure 5a). The maximum firing rate reached during the response to the 10^th^ pulse was only 39% that of the 1^st^ pulse.

At higher pulse rates, 4 and 10 pps, the Ph-ORNs still showed responses to individual pulses (Figures 4a and 5a) but the maximum firing frequency reached during peaks of activity was lower compared to 1 pps. There was a significant effect of repetition rate on the maximum firing rate reached in response to each pulse (ANOVA, *P* = 5.2 e^−15^). Furthermore, the reduction of firing activity between first pulse and successive pulses was more evident compared to 1 pps (Figure 5a). The percentage of decrease of the maximum firing rate reached during the responses to the 10^th^ pulse compared to the first pulse was 86% at 4 pps and 84% at 10 pps. In addition, the minimum firing rates between peaks was significantly higher at 4 pps and 10 pps compared to 1 pps (ANOVA, *P* = 2.2e^−16^).

### 4 – General effects of linalool

Linalool itself does not activate the Ph-ORNs [11]. During a 4 s application of linalool background the mean firing rate of Ph-ORNs was 1.7±0.4 spikes/s (mean ± SEM) and not significantly different to before linalool presentation, 1.6±0.4 spikes/s (Student's test, *P* = 0.80). In a linalool background applied 0.5 s before the first pulse of pheromone, Ph-ORNs still followed the pheromone pulses (Figures 4b and 5b). However, the maximum firing rates to pheromone pulses were significantly decreased at all repetition rates (ANOVA, *P* = 6.61 e^−07^) (Figure 5b). Furthermore, the Ph-ORN firing activity between pulses at 1 and 4 pps in a linalool background dropped to a lower level compared to that in a neutral background (ANOVA, *P*<0.0001; Figure 5, a and b).

Since peaks of responses appeared shorter in linalool background (Figure 5b), we compared the speed of decay determined from a sample of 54 responses to the first pulse in clean air and 63 in linalool background to determine whether linalool affected the kinetics of decay. The fall times were not compared at the other repetition rates, because the inter-pulse intervals were too small to estimate reliably the exponential decay. After standardization of the maximum firing amplitude to 100, the slope of the firing decay in linalool background ([eq 2], c = 2.66) and in a neutral background (c = 2.605) were very similar. Correspondingly, the estimated 90% decay time of firing frequency in linalool background (161 ms) and in a neutral background (170 ms) were almost equivalent (Figure 6). Thus, most of the conspicuous change in thickness of the peak cannot be attributed to an alteration in the Ph-ORN recovery.

### 5 – Temporal analyses

To better assess the effects of a linalool background on the temporal coding by Ph-ORNs, we compared the firing responses in neutral or linalool backgrounds by calculating the local contrast (firing rate at maximum in relation to minimum in dB), calculating the ability of Ph-ORNs to follow pulse rates (LSP), and measuring the information content of the sensory output (Jensen Shannon divergence).

**Figure 7:**
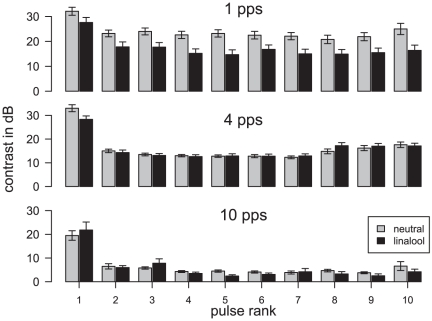
Linalool background maintains the contrast within a pulsed pheromone stimulation. To determine the ability of Ph-ORN to code the temporal pattern of pulsed stimulus we calculated the contrast in dB between minimum and maximum firing rates as C = 20 * log_10_ (max/min) at 1, 4 and 10 pps (same data as in Figure3). Mean contrast for individual peaks of responses to pheromone pulses in linalool (black bars) or neutral (grey bars). Error bars: SEM.

The contrast was slightly lower for pulses inside a pulse train (ANOVA, *P* = 2.8e^−6^), compared to the first pulse, and in presence of linalool, compared to neutral background at 1 pps (*P* = 2.6e^−16^) (Figure 7). At 4 pps, the contrast was lower compared to 1 pps (*P* = 2.2e^−16^), but similar in a neutral background and in linalool (*P* = 0.54). At both repetition rates, and for every pulse, the contrast was always above 12 dB, enabling clear discrimination of pulses among background firing activity. At 10 pps, most of the peaks were not visible any more and contrast dropped below 5 dB.

The Lomb and Scargle period analysis revealed that at 1 pps 80% of the Ph-ORNs followed the stimulus pattern in neutral background versus 67% in linalool; the difference was not significant (χ^2^ test = 1.85, *P* = 0.17). At 4 pps, significantly less Ph-ORNs followed the stimulation pattern in neutral (29%) than in linalool background (51%) (χ^2^ test = 4.19, *P* = 0.04). No Ph-ORN followed the 10 pps stimulation pattern neither in air nor in linalool.

**Figure 8:**
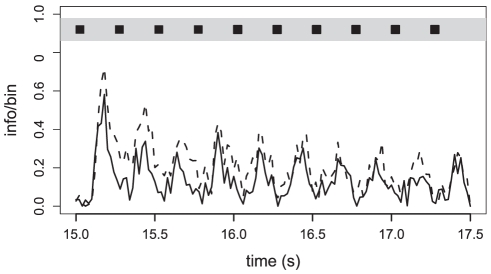
Linalool background improves the information content of the firing activity of a population of Ph-ORNs in response to pulsed pheromone. The Jensen Shannon divergence (D_JS_) was calculated bin by bin (bin size = 20 ms) between control and pheromone pulses in air (n = 57) or between control and pulses in linalool (n = 46). The info/bin value varies from 0 (the probability function of spike distribution in control and stimulated neurones are identical) to 1 (the probability distributions do not overlap). Dashed line: D_JS_ curve for pheromone pulses in air; solid line: D_JS_ curve for pheromone pulses in linalool.

Since the perception of information depends on the processing of the activity of large populations of ORNs in the central nervous system, we calculated the Jensen Shannon divergence (D_JS_) of neuronal activities in response to a train of pheromone pulses at 4 pps in neutral and linalool backgrounds. For both background conditions, the D_JS_ was calculated between recordings in pheromone stimulated and control (no pheromone pulses) situations to obtain a measure of the information flux. At a repetition rate of 4 pps in neutral background, the information flux followed the pulse train showing maxima during pulses and minima between pulses (Figure 8). However, the D_JS_ were still high during the inter-pulse intervals indicating high information content within the activity of Ph-ORN population. In linalool background, the maximum information flux still reproduced the stimulus periodicity although it was slightly lower compared to the neutral background. However the information content decreased faster after each pulse and during the inter-pulses it was not different from pre-stimulus firing activity.

## Discussion

### A linalool background reduces the responses to the pheromone of ORNs and affects orientation behaviour

The most conspicuous effect of the linalool background was to reduce the maximum firing rate of Ph-ORNs in response to a pheromone pulse. This effect was observed for all the pulses within a pulse train, and it contributed to a reduction in sensitivity during pulse trains at low repetition rates. The pheromone odorized flow was kept constant in behavioural experiments and, although the PID signal showed some fluctuations in the concentration of pheromone above the sphere, those fluctuations were less intense than those resulting from a pulsed stimulation. Thus, although the stimulations conditions in behavioural and electrophysiological experiments were not exactly the same, for male searching for a pheromone source the presence of linalool should globally result in a reduction of the perceived concentration of pheromone. Correspondingly, the male orientation behaviour to the pheromone source became temporally less focused at the onset of a linalool background. The orientation behaviour quickly recovered, although the linalool background was maintained, showing that a sudden change of the stimulus intensity might be more important than its absolute value. Such a decrease of sensitivity might even have an advantage close to the source of pheromone by preventing Ph-ORN from saturation and might also increase the dynamic range of the pheromone detection system.

### Linalool sharpens the responses of ORNs

As shown previously in preceding works, Ph-ORNs were able to fire in synchrony with pulsed stimulations. However the responses were almost fused at 10 pps. We found that the 90% decay time of the firing response was 170 ms, a value only slightly above the 90% decay of the stimulus measured with the PID (150 ms). This is likely the reason why at 4 pps, (with 200 ms inter-pulse intervals) responses to individual pulses were not completely separated. Thus, in our set–up, the hysteresis of the stimulation device must have been the most determinant limit to temporal resolution, the PID measurements showing a progressive decrease of the linalool concentration after closing of the electro-valve. The responses of insect ORNs are generally considered as phasic tonic. Long recovery times, 0.96 and 1.92 s, were observed in two ORN types of female *Bombyx mori* [21] for instance. However, although stimulus delivering systems using fast electro-valves may produce square pulses of odorized air, the kinetics of the odour concentration around the antenna is slower as shown by our PID measures. Differences in the experimental set-ups used for stimulation may be at the origin of the great differences between the values reported in the literature for the temporal resolution of insect ORNs (from 5 [15], to 40 pps [20]). Interestingly, it might result that the use of olfactory stimulators specially designed to achieve fast onset and offset of the olfactory stimulus will reveal that capacity of Ph-ORNs to respond to fast change of odorant concentration are even faster as found so far.

Linalool had a sharpening effect, the peaks of firing response to pheromone pulses being narrower in a linalool background and the 90% decay time being shorter, but the slope was not altered. Our PIDs measures showed that odorants are still present in the permanent air flow bathing the antenna during the measured decay time of the firing response. Thus, it is likely that linalool indirectly affects the response decay by impeding these residual pheromone molecules to reach or bind to their receptors as postulated by Party et al [11]. This seems to exclude a direct effect of linalool on the cellular or molecular mechanisms of response termination. In particular, this excludes an interference of linalool with odorant degrading enzymes (ODEs), such as esterases, which are postulated to eliminate pheromone molecules from the sensilla after their binding to olfactory receptors [37], [38].

### The coding of high repetition rates is also limited by the recovery from sensory adaptation

The responses to pheromone pulse trains showed a decrease in firing response to individual pulses according to pulse rank. A similar decline was observed by Barrozo and Kaissling [21] who evidenced that consecutive responses were lower than the first response at pulse intervals smaller than 1.92 s [21]. The response reduction cannot be explained by an exhausting of the pheromone source because the PID signal with linalool or other odorants did never show a decrease in concentration of the odorant (unpublished data). Since the interval between pulses at 1 pps is greater than the time necessary for the Ph-ORNs to recover, the reduction of amplitude should be interpreted as a true sensory adaptation. Adaptation is a stimulus-induced decrease of responsiveness involving an alteration in the transduction pathway that presents different forms [25]. Pulse trains may cause short term adaptation induced by the 2^nd^ pulse and cumulative adaptation (depending on the repetition rate) [24]. Short term adaptation and response decline kinetics contribute to limit the capacity of ORNs to follow high repetition rate stimulation. By reducing the maximum firing rate, adaptation of ORNs is a limiting factor to follow the stimulus pattern in our experiments. In such a case, the presence of a linalool background might partially compensate for the effects of adaptation. Decreased firing activity between pulses in the presence of linalool possibly compensated for reduced maximum firing rate as it is visible in contrast calculations of ORN coding of changes in concentration during and between stimulus pulses. The contrast was preserved in presence of the odorant background.

### Consequences on temporal coding by the Ph-ORN population

The main effect of linalool background was to reduce the number of spikes emitted by the Ph-ORNs, which results in a loss of sensitivity to the insect. However, with respect to the detection of the intermittency of the odorant signal, the relative changes of firing frequency within a population of neurones are probably more critical than the absolute level of activity of individual neurones. As clearly visible on figure 4, individual Ph-ORNs showed differences in their ability to follow stimulus pulses. As seen at higher pulse rate, firing rate was still elevated during inter pulses intervals. Measured information between the pulses was still very high and the peaks were less marked. Reduction of the ORN firing rate in linalool background did thus not have major impact on the information flow. Our analyses showed that information flow more accurately followed the individual pulses. Information about stimulus intermittency was thus very well preserved over the population of Ph-ORNs in a noisy environment.

### Conclusions

Animals responding to a specific olfactory signal in an odorant background are faced with sensory adaptation to the signal and masking by noise. It is expected that both phenomena contribute to degrade the coding of the specific signal by ORNs in a synergistic way. However, relatively to the rapid fluctuations in the signal that are in a sub-second range, adaptation has long-lasting effects because it affects transduction pathways for several seconds. In turn, masking of the signal by a background odour has transient effects that rapidly vanish once the presentation of the non-specific compound is over. We have shown that due to the reduced response rate both during and between pulses, an odorant background ensures preservation of temporal parameters of a specific olfactory signal, independently of effects on response intensity. One can thus postulate that a natural background will preserve the temporal structure of a signal-odour plume, which is necessary for orientation to odour source. Furthermore, the reduction in intensity of the response might be favourable at high levels of pheromone concentration by preventing the saturation of Ph-ORNs. This partly explains why moths show such remarkable ability to orient to pheromone sources, even in highly changing environments. While it seems attractive to use natural odorants as masking odours for the control of insects by disrupting pheromone communication with environmentally safe agents, our findings underline the complexity of mechanisms involved in masking by linalool and plead for more investigations on the interactions between odorants.
